# Use of a mitochondrial COI sequence to identify species of the subtribe Aphidina (Hemiptera, Aphididae)

**DOI:** 10.3897/zookeys.122.1256

**Published:** 2011-08-11

**Authors:** Jian-Feng Wang, Li-Yun Jiang, Ge-Xia Qiao

**Affiliations:** 1 Key Laboratory of Zoological Systematics and Evolution, Institute of Zoology, Chinese Academy of Sciences, No. 1 Beichen West Road, Chaoyang District, Beijing 100101, P.R. China; 2 Liaoning Key Laboratory of Urban Integrated Pest Management and Ecological Security, Shenyang University, Shenyang 110044, P. R. China

**Keywords:** Hemiptera, Aphidinae, Aphidina, mitochondrial COI gene, intraspecific divergenuce, interspecific divergence, identification

## Abstract

Aphids of the subtribe Aphidina are found mainly in the North Temperate Zone. The relative lack of diagnostic morphological characteristics has hindered the identification of species in this group. However, DNA-based taxonomic methods can clarify species relationships within this group. Sequence variation in a partial segment of the mitochondrial *COI* gene was highly effective for identifying species within Aphidina. Thirty-six species of Aphidina were identified in a neighbor-joining tree. Mean intraspecific sequence divergence in Aphidina was 0.52%, with a range of 0.00% to 2.95%, and the divergences of most species were less than 1%. Mean interspecific divergence within previously recognized genera or morphologically similar species groups was 6.80%, with a range of 0.68% to 11.40%, with variation mainly in the range of 3.50% to 8.00%. Possible reasons for anomalous levels of mean nucleotide divergence within or between some taxa are discussed.

## Introduction

Aphids are globally important invasive agricultural pests (Foottit et al. 2006; Messing et al. 2007). The aphid subtribe Aphidina (Hemiptera: Aphididae: Aphidinae) contains approximately 670 described species worldwide (Remaudière and Remaudière 1997). Several of them are on the list of the most important agricultural pests, including *Aphis gossypii* Glover, *Aphis glycines* Matsumura, *Schizaphis graminum* (Rondani) and *Toxoptera citricidus* (Kirkaldy) (Blackman and Eastop 2000). The largest aphid genus *Aphis* includes 550 species, approximately 10% of the total number of known aphid species; the nominal subgenus *Aphis* contains more than 90% of these species.

Resolving species relationships within Aphidina has been hindered by the lack of variation in morphological features. In particular, species of *Aphis* lack diagnostic morphological characteristics. Although some species can be easily distinguished by a single diagnostic morphological trait, many of them cannot be separated morphologically. Consequently, many species have been grouped according to their gross morphological similarities. The resultant entities, known as “groups of species”, have no taxonomic validity because they simply contain species that are difficult to tell apart morphologically.

Three such “groups of species”, the black-backed, black and *frangulae*-like groups, have been described in Europe (Heie 1986; Stroyan 1984). Each of these groups is based on its similarity to a single polyphagous or oligophagous species, *Aphis craccivora*, *Aphis fabae* and *Aphis frangulae*/*gossypii*,respectively. Within these groups, it is still difficult to accurately identify some taxa (Coeur d’acier et al. 2007). Zhang and Zhong(1981) dealt with Chinese members of the *Aphis craccivora* complex, and divided them into six species and subspecies, *Aphis sophoricola* Zhang, *Aphis atrata* Zhang, *Aphis craccivora craccivora* Koch, *Aphis craccivora usuana* Zhang, *Aphis robiniae robiniae* Macchiati and *Aphis robiniae canavaliae* Zhang; however, Remaudière and Remaudière (1997) merged these six taxa into a single species, *Aphis craccivora* Koch. Zhang et al. (2010) studied the subspecies differentiation of *Aphis fabae* Scopoli based on morphological and genetic data analysis.

Because the identification of aphid species is often based on presumed host plant specificity, polyphagous or oligophagous species, such as *Aphis craccivora*, *Aphis fabae* and *Aphis frangulae*/*gossypii*, can easily be misidentified (Coeur d’acier et al. 2007). These types of taxonomic problems are universal in Aphididae. Therefore, the development of an accurate and quick method to identify aphids is essential for the timely detection of new invasive species and the prevention of severe crop losses.

A standard region of the mitochondrial gene that encodes cytochrome c oxidase I (*COI*) was originally used to identify unknown aphid specimens (Hebert et al. 2003a). Although its taxonomic utility is limited by the requirement for a complete database of voucher specimens which individuals can be compared with (Moritz and Cicero 2004; Will and Rubinoff 2004), DNA barcoding has nonetheless proven a useful tool for taxonomists (Schindel and Miller 2005). DNA taxonomy has successfully identified cryptic species in a diverse range of taxa (Brown et al. 2003; Ball and Hebert 2005; Barrett and Hebert 2005; Foster et al. 2004; Hogg and Hebert 2004; Monaghan et al. 2005; Cardoso and Vogler 2005; Ward et al. 2005; Vences et al. 2005; Clareet al. 2007) and has been especially useful for identifying aphid species (Foottit et al. 2008; Wang and Qiao 2009).

In this paper, we attempt to clarify some of the current taxonomic confusions and previously obscure species relationships within Aphidina. The variation in a short mitochondrial *COI* gene sequence that we found was used. Its utility was assessed as a method to accurately and quickly identify an assemblage of mainly Chinese aphids at the species level.

## Materials and methods

### Taxon sampling and data collection

We examined 198 *COI* sequences; 143 sequences were extracted from Chinese samples, and 55 sequences of European and North American samples were downloaded from GenBank. The ingroup included 176 Aphidina specimens from 36 species (subspecies) (34 species after revision) and 9 genera (subgenera). The outgroup was composed of 22 sequences from 5 species of 2 genera in the sister subtribe Rhopalosiphina (6 sequences), 7 species of 6 genera in Macrosiphini (12 sequences); and 4 species of 4 genera in Fordinae (4 sequences).

Three subgenera of the genus *Aphis* from the Palaearctic region were represented in our samples, namely *Aphis*, *Bursaphis* and *Protaphis*. Two recognized major “groups of species”, the black-backed and *frangulae*-like aphids, and some morphologically distinct species (including *Aphis spiraecola*, *Aphis nerii* and *Aphis farinosa*) were also examined.

Collection information for all samples, including locations, host plants and collection dates, are shown in Appendix 1. Except for specimens for slide-mounting that were stored in 70% ethanol, all other specimens were stored in 95% or 100% ethanol. All samples and voucher specimens were deposited in the National Zoological Museum of China, Institute of Zoology, Chinese Academy of Sciences, Beijing, China.

About 143 samples including many individuals, one to three individuals per sample were isolated DNA for molecular studies, and three to five individuals per sample were made to slide-mounted specimens for morphological examination. Voucher specimens of all samples were identified from their main morphological diagnostic features, and compared with previously identified specimens. The species name of each sample has been provided in Appendix 1. *Aphis asclepiadis* Fitch was identified from the description of Zhang and Zhong (1983), and by comparison with their specimens.

### DNA extraction, PCR and sequencing

Total DNA was isolated from one to three individuals per sample, followed by a standard phenol-chloroform-isoamylalcohol (PCI) extraction with some modifications (Sambrook et al. 1989). Polymerase chain reaction (PCR) was used to amplify a 1250–1300 base pair (bp) segment of the mitochondrial *COI* gene. There were two primer pairs, including two forward primers and one reverse primer. LCO1718 (Simon et al. 1994) was one of the forward primers. We designed the other forward primer (5'-TATATCTTTCCCACGATTAAATAA-3') and the reverse primer (5'-GCATATTAATTCTGCCATATTAG-3'). Each PCR contained 5 µL of 10× PCR buffer at pH 8.3 (10 mmol/L of Tris HCl at pH 8.3, 1.5 mmol/L of MgCl2, 50 mmol/L of KCl, 0.01% NP-40), 1 µL of 10 pmol/L of each dNTP (C, G, A, T) (Takara Biotech, Dalian, China), 2 µL of 10 *μ*mol/L of each primer, 1.0 U (1 U≈16.67 nkat) of Taq DNA polymerase (Sangon Biotech, Shanghai, China) and 2 µL of the DNA template. The reaction was performed using a GeneAmp PCR System 9700 (Applied Biosystems, USA) under the following conditions: 95°C for 5 min; 35 cycles at 94°C for 1 min, 48–54°C for 1 min, 72°C for 1 min; and a final extension step of 10 min at 72°C. These two primer pairs worked well for all species examined.

Sequencing reactions were performed with the corresponding amplifying primers from both directions using a BigDye Terminator Cycle Sequencing Kit v.2.0 (Applied Biosystems, USA) and run on an ABI 3730 automated sequencer (Applied Biosystems, USA).

### Assembling and aligning sequences

To obtain single consensus sequences, chromatograms, including sense and antisense, were analyzed and assembled using the Seqman module of the DNAStar* 5.0 software package (DNASTAR, Inc.1996). We checked the accuracy of the nucleotide sequences by confirming that they could be translated into proteins by using Editseq (DNASTAR, Inc. 1996). Sequences were deposited in GenBank under Accession Nos. FJ965596–FJ965749.

### Aphid species’ COI profiles

*COI* profiles were obtained from a neighbor-joining (NJ) tree with Kimura-2-parameter (K2P) distances created using MEGA3.1 (available at http://www.megasoftware.net). The K2P model provides the best metric when genetic distances are low (Nei and Kumar 2000). We used a simple NJ algorithm to identify species based on sequence similarity rather than reconstruct deeper phylogenetic relationships using NJ analysis.

### Data analysis

The sequences were manually aligned with the Bioedit sequence editor (Hall 1999), and the alignment was subsequently pruned to 1145 bp. To obtain a more comprehensive range of *COI* sequences, we combined the 591 bp *COI* sequences from Chinese specimens with the downloaded sequences. Nucleotide-sequence divergences were calculated using Kimura’s two-parameter model (Kimura 1980). Based on K2P distances, we obtained intraspecific sequence divergences for all species sequences from more than two individuals. When a single species had sequences from several individuals, one sequence was randomly chosen to represent that species. K2P divergences for all congeneric species pairs were studied and plotted as a frequency histogram. The mean intra- and interspecific K2P divergences were calculated as the overall mean of all pairwise comparisons within each species and genus, respectively.

## Results

### Data analysis

Including outgroups, 52 species were identified among the 198 taxa examined. Of the 591 bp that were analyzed, 373 were conserved, 218 were variable and 191 were parsimony-informative; 404 sites were constant, 187 were variable and 164 were parsimony-informative for ingroups only. These sequences were heavily biased toward A and T nucleotides (means: T = 39.4%, C= 13.9%, A = 34.5%, G = 12.2%).

### Taxonomic assignments and NJ tree structure

In the NJ tree (Fig. 1a–d), most of the aphid species and subspecies included in our NJ profile possessed a distinct *COI* sequence. Although the *Cryptosiphum artemisiae* Buckton clustered with Macrosiphini and *Swirskiaphis bambuciepula* Zhang was embedded within Rhopalosiphina, all other Aphidina specimens formed a cohesive group. The Rhopalosiphina appear to be a monophyletic sister group to Aphidina, whereas the Macrosiphini and Fordinae are rooted and clustered, separately.

Within Aphidina, two subgenera (*Bursaphis* and *Protaphis*) and the three recognized “groups of species” within the subgenus *Aphis* all formed separate, cohesive clusters. However, the species from the subgenus *Aphis* did not cluster together but were divided into the following six main clades: ‘black-backed species’, ‘black species’ ‘*frangulae*-like species’, ‘*spiraecola*-like species’, *Aphis nerii* and *Aphis farinosa*. *Aphis nerii* and *Aphis asclepiadis* (=*Aphis nerii*) cluster together, and with lower nucleotide divergence (0.00%–0.17%).

**Figure 1a.**
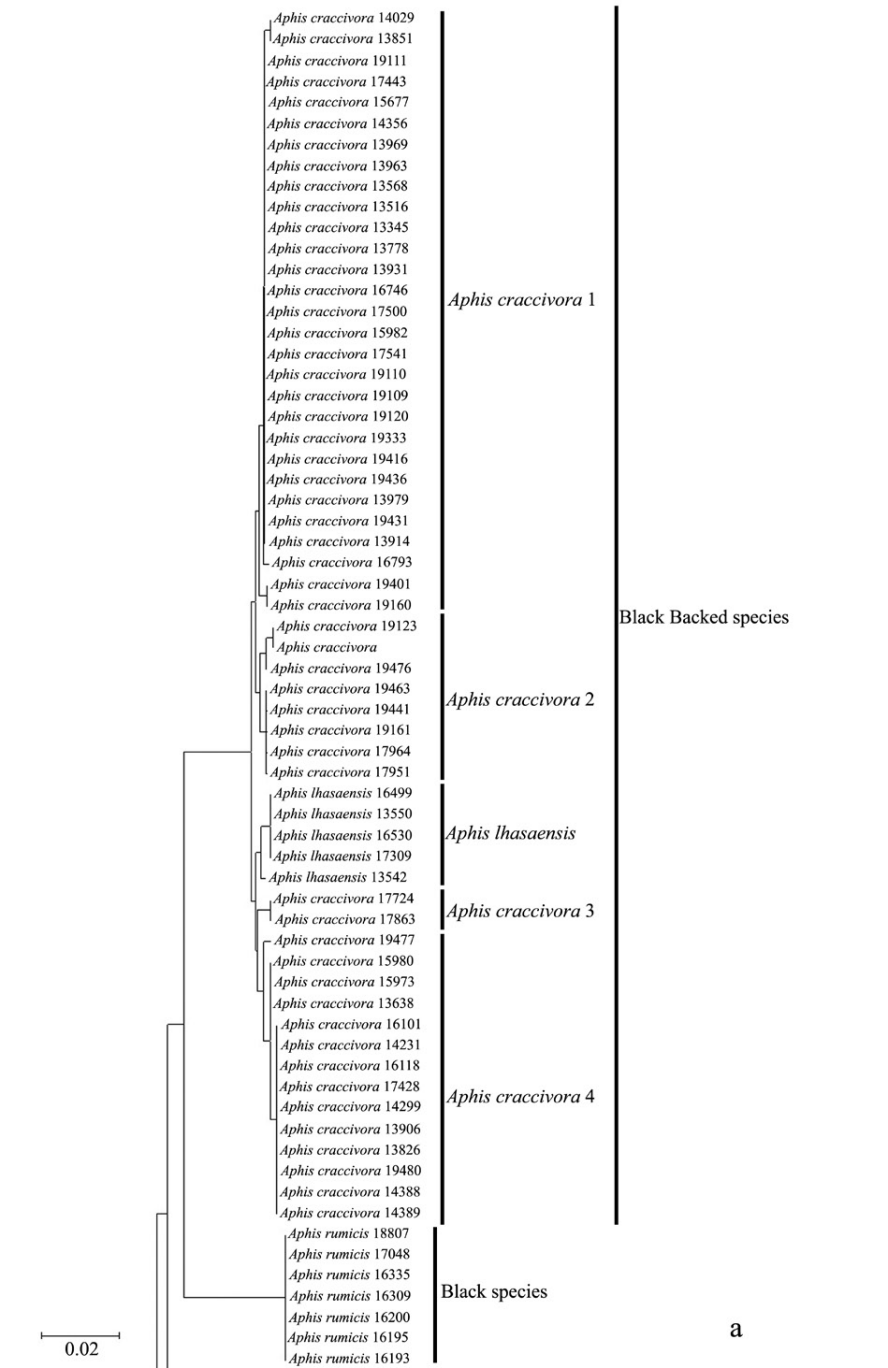
Neighbor-joining analysis of 198 specimens. It was based on *COI* sequence divergence in 591 bp of the *COI* gene using Kimura’s two parameter model.

**Figure 1b.**
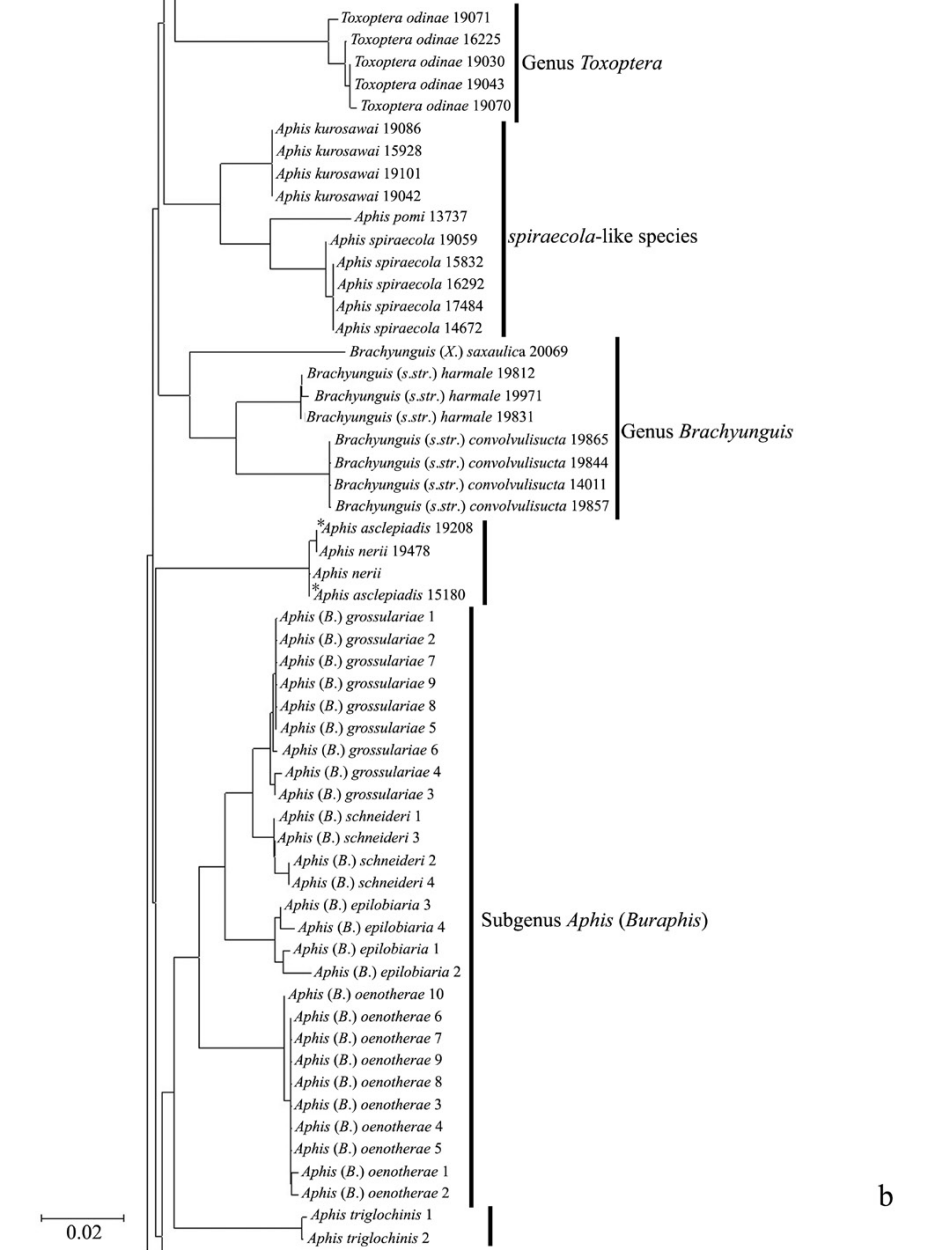
Neighbor-joining analysis of 198 specimens. It was based on *COI* sequence divergence in 591 bp of the *COI* gene using Kimura’s two parameter model. * They were originally misidentified as *Aphis asclepiadis* Fitch; actually, should be *Aphis nerii* Boyer de Fonscolombe.

**Figure 1c.**
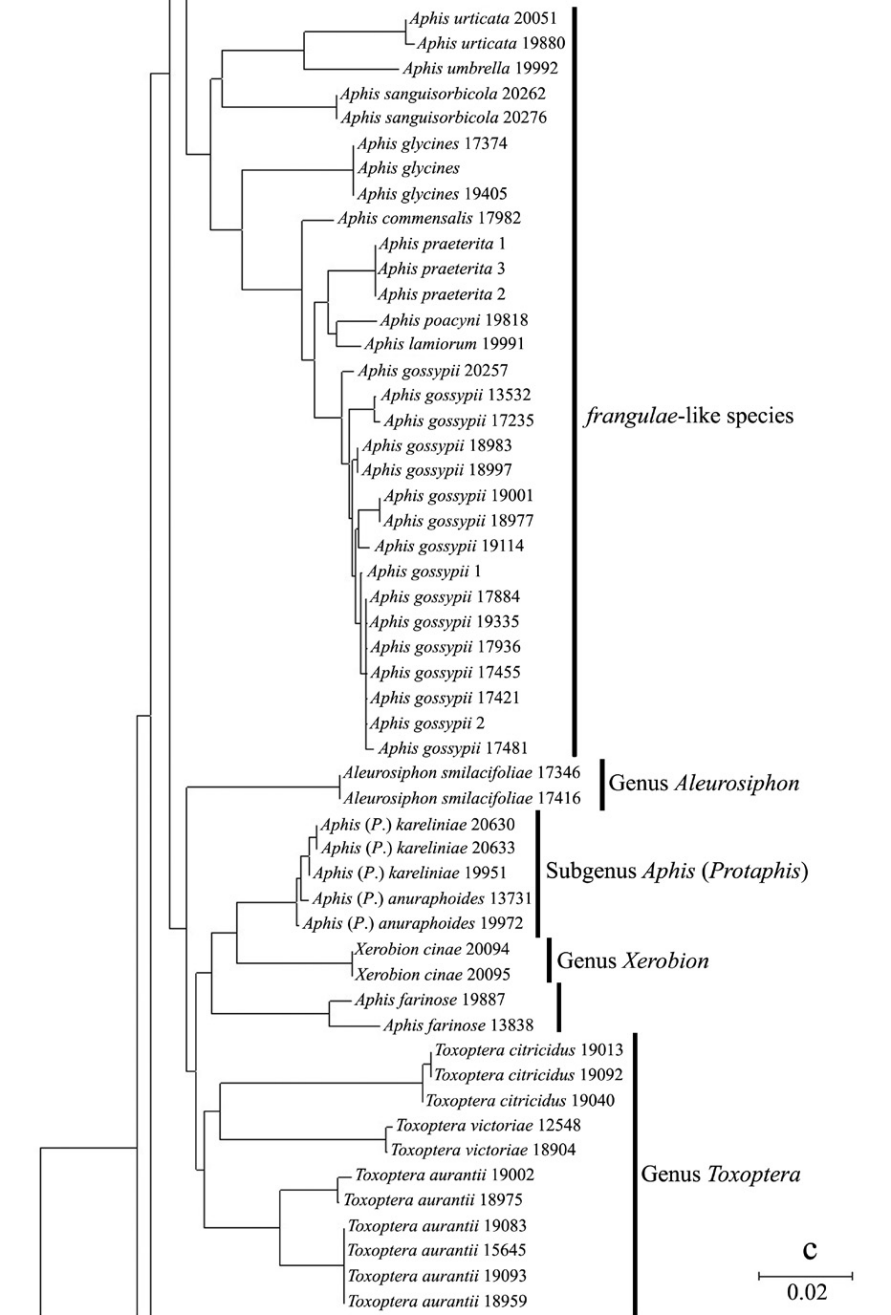
Neighbor-joining analysis of 198 specimens. It was based on *COI* sequence divergence in 591 bp of the *COI* gene using Kimura’s two parameter model.

**Figure 1d.**
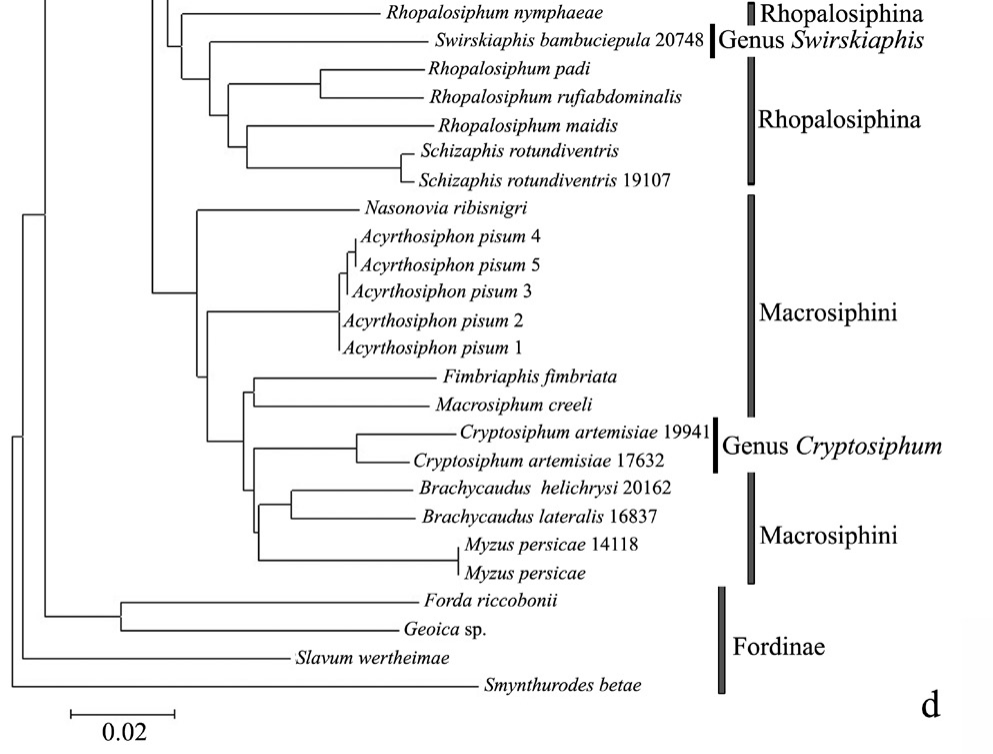
Neighbor-joining analysis of 198 specimens. It was based on *COI* sequence divergence in 591 bp of the *COI* gene using Kimura’s two parameter model.

The genus *Toxoptera*, represented in our sample by sixteen individuals from four species, did not cluster together but was separated into three clades. Although six individuals of *Toxoptera aurantii* formed a cohesive group, two subgroups were apparent.

### Nucleotide diversity

Among 19,503 pairwise combinations in 198 specimens, the mean *COI* divergence was 6.99%, with a range of 0.00% to 17.56%, and mostnucleotide divergence ranged from 0.00% to 1.75% and 3.75% to 13.00%.

**Figure 2.**
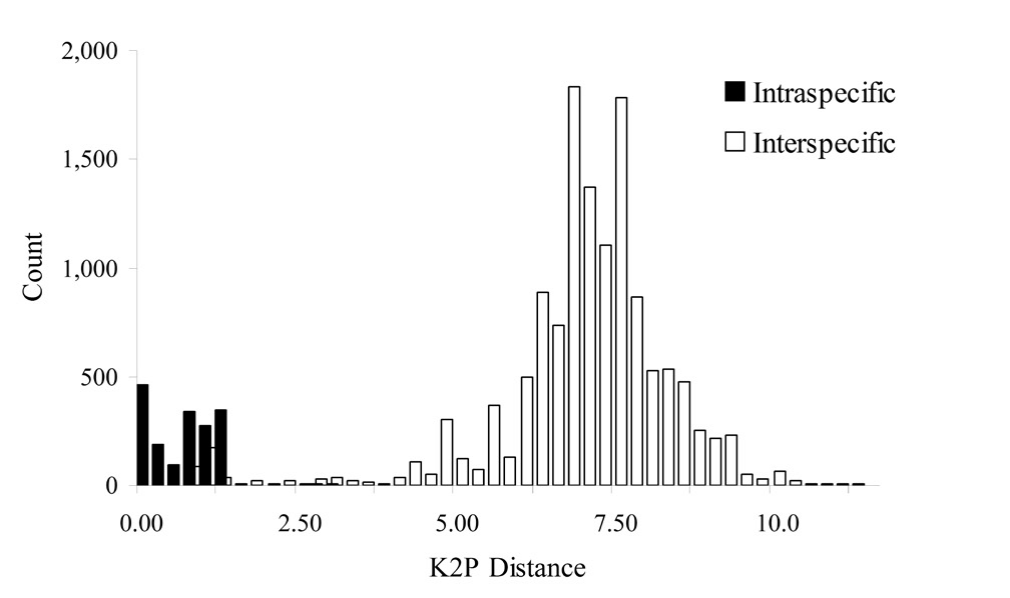
Histogram of intra- and interspecific nucleotide divergence in Aphidina. Divergences were calculated by using Kimura’s two parameter (K2P) model.

Omitting *Cryptosiphum artemisiae* and *Swirskiaphis bambuciepula*, the mean *COI* divergence among the 14,878 species pairs placed within Aphidina by Remaudière and Remaudière (1997) was 6.10%, with a range of 0.00% to 11.40% (Fig. 2). These results indicate that the average intraspecific sequence divergence in Aphidina is 0.52%, with a range of 0.00% to 2.95% (Table 1), and the average interspecific divergence is 6.80%, with a range of 0.68% to 11.40% (Table 2).

**Table 1.** Mean and range of intraspecific nucleotide divergences for Aphidina species. Data were estimated by using Kimura’s two parameter model.

Species	No. of individuals	Mean percentdivergence %	Range %	SD %
*Aphis craccivora*	53	0.55	0.00–1.20	0.40
*Aphis craccivora* 1*	29	0.08	0.00–0.51	0.13
*Aphis craccivora* 2*	6	0.26	0.00–0.51	0.23
*Aphis craccivora* 3*	2	0.00	0.00	/
*Aphis craccivora* 4*	14	0.12	0.00–0.51	0.16
*Aphis lhasaensis*	5	0.14	0.00–0.34	0.18
*Aphis farinosa*	2	1.54	1.54	/
*Aphis glycines*	3	0.00	0.00	0.00
*Aphis gossypii*	16	0.53	0.00–1.37	0.39
*Aphis kurosawai*	4	0.11	0.00–0.17	0.00
*Aphis asclepiadis*	2**	0.17	0.17	/
*Aphis nerii*	2	0.17	0.17	/
*Aphis praeterita*	3	0.20	0.00–0.51	0.26
*Aphis rumicis*	7	0.00	0.00	0.00
*Aphis sanguisorbicola*	2	0.00	0.00	/
*Aphis spiraecola*	5	0.07	0.00–0.17	0.09
*Aphis triglochinis*	2	0.17	0.17	/
*Aphis urticata*	2	0.17	0.17	/
*Aphis (Bursaphis) epilobiaria*	4	0.82	0.34–1.19	0.33
*Aphis (Bursaphis) grossulariae*	9	0.14	0.00–0.51	0.14
*Aphis (Bursaphis) oenotherae*	10	0.10	0.00–0.34	0.11
*Aphis (Bursaphis) schneideri*	4	0.23	0.00–0.34	0.18
*Aphis (Protaphis) anuraphoides*	2	0.17	0.17	/
*Aphis (Protaphis) kareliniae*	3	0.11	0.00–0.17	0.10
*Aleurosiphon smilacifoliae*	2	0.00	0.00	/
*Brachyunguis convolvulisucta*	4	0.00	0.00	0.00
*Brachyunguis harmalae*	3	0.11	0.00–0.17	0.10
*Cryptosiphum artemisiae*	2	2.95	2.95	/
*Toxoptera aurantii*	6	1.50	0.00–2.95	1.41
*Toxoptera citricidus*	3	0.11	0.00–0.17	0.10
*Toxoptera odinae*	5	0.37	0.00–0.85	0.29
*Toxoptera victoriae*	2	0.17	0.17	/
*Xerobion cinae*	2	0.00	0.00	/
All	169	0.52	0.00–2.95	0.44

*intraspecific clades.** The two samples were originally misidentified as *Aphis asclepiadis* Fitch; actually, they should be *Aphis nerii* Boyer de Fonscolombe.

**Table 2.** Interspecific nucleotide divergences for species in 9 genera or “groups of species” in Aphidina. Data were estimated by using Kimura’s two parameter model.

Groups	No. of species (individuals )	Mean percentdivergence %	Range %	SD %
Black backed species	5* (58)	0.83	0.51–1.20	0.18
Black backed species	2 (58)	0.80	0.68–1.20	0.09
spiraecola-like species	3 (10)	3.85	3.31–4.55	0.32
frangulae-like species	9 (30)	4.90	1.20–8.91	2.16
*Aphis (Protaphis)*	2(5)	0.54	0.34–0.68	0.13
*Aphis (Buraphis)*	4 (27)	3.40	0.85–4.41	1.05
*Brachyunguis*	3 (8)	5.01	3.84–6.97	1.49
*Toxoptera*	4 (11)	6.83	2.59	2.11
All (revised)	32* (173)	6.80	0.68–11.4	1.45

* No. of clades.

## Discussion

As expected from previous DNA studies of aphids (Shufran et al. 2000, Anstead et al. 2002, Favret and Voegtlin 2004, von Dohlen 2000, von Dohlen et al. 2002, 2006, Zhang and Qiao 2007a, b, Foottit et al. 2008, Kim and Lee 2008, Wang and Qiao 2009), the DNA sequences we used were heavily biased towards the nucleotides A and T. Nonetheless, we found that the variation in a partial segment of the mitochondrial *COI* gene can be used to identify species within Aphidina.

### Sequence divergences within and among species

The magnitude of *COI* divergence varies among different animal groups. Hebert et al. (2003b) found that *COI* divergence among 13,320 species in 11 families ranged from 0.0% to 53.7%. Most pairs (79%) showed over 8% sequence divergence, and most species pairs (98%) had over 2% sequence divergence. Our data indicate that the mean *COI* divergence within Aphidina is 6.10%, with a range of 0.00% to 11.40%.

This degree of intraspecific divergence is similar to that found in other animal taxa. For example, treating the provisional species as separate taxa, the 0.52% intraspecific variation we found in Aphidina is comparable to values reported for other taxa, such as 0.27% in North American birds (Hebert et al. 2004b), 0.39% in marine fish (Ward et al. 2005), an average value of 0.60% in Guyanese bats (Clare et al. 2007), 0.46% in Lepidoptera (Hajibabaei et al. 2006), 0.11% in North American mayflies (Ball et al. 2005) and 0.14% in spiders (Barrett and Hebert 2005).

The six specimens assigned to the species *Toxoptera aurantii* (Boyer de Fonscolombe) were divided into two different clades, with divergences ranging from 2.59% to 2.95%, far higher than those between the other Aphidina species. This result indicated that *Toxoptera aurantii* probably contains cryptic species, but we did not find any distinct morphological differences after checking the specimens. These results were consistent with the conclusions of Wang and Qiao (2009).

Omitting *Cryptosiphum artemisiae*, which should be moved into the tribe Macrosiphini (Kim and Lee 2008), and *Swirskiaphis bambuciepula* (see below), the mean sequence divergence among Aphidina species was 6.80%. This divergence is similar to those reported in other animal groups, for example, 7.93% in North American birds (Hebert et al. 2004b), 9.93% in marine fish (Ward et al. 2005), an average value of 7.8%±4.78% in Guyanese bats (Clare et al. 2007) and 4.58%, 4.41% and 6.02% in Lepidoptera (Hajibabaei et al. 2006).

However, mean divergences among the black-backed “groups of species” and the two species of *Aphis (Protaphis)* ranged from 0.34% to 0.85%, far lower than that between the other Aphidina groups, and the divergence between *Aphis (Bursaphis) grossulariae* Kaltenbach and *Aphis (Bursaphis) schneideri* (Börner) was also unusually low (0.85%).

### Systematic status of some taxa

Pairwise *COI* sequence divergence among congeneric animal species is generally over 2% (Hebert et al. 2003b). Lower interspecific divergences are unusual in other animal groups but are often found in Aphidina. For instance, some clearly distinct species in the genera *Aphis* and *Illinoia* exhibited divergences of less than 1% (Foottit et al. 2008).

**Evidence for host races within *Aphis craccivoraa***

In our NJ tree, *Aphis craccivora* was divided into four clades and clustered together with *Aphis lhasaensis* Zhang. Pairs of clades in this group had *COI* sequence divergences ranging from 0.51% to 1.20% (mean=0.83%), lower than the interspecific divergences of the other aphids. *Aphis lhasaensis* was found in Tibet, infesting subterranean parts of *Astragalus sinicus* L. (Fabaceae). It is similar to *Aphis craccivora*, except that its abdominal segments II–IV have large marginal tubercles. Our results suggest that *Aphis lhasaensis* should be regarded as a Tibetan subspecies of *Aphis craccivora* rather than a separate species. Two of the four clades of *Aphis craccivora* showed some evidence of association with different host plants in the same family Fabaceae, indicating the presence of host-adapted races in Chinese populations of this species. In particular, 13 out of 14 samples collected from *Robinia pseudocacia* were of clade 1, and 12 out of 22 samples of *Sophora japonica* were of clade 4.

**The Chinese record of *Aphis asclepiadis* Fitch should be referred to *Aphis nerii* Boyer de Fonscolombe**

Zhang and Zhong (1983) recorded *Aphis asclepiadis* Fitch in China, and on the basis of their record and description, two samples were identified as this species. The *COI* sequence of these samples was found to be similar or identical to that of *Aphis nerii*; and on subsequent re-examination of slide-mounted specimens, they were identified as this species, which feeds on both Asclepiadaceae and Apocynaceae. The record of *Aphis asclepiadis* Fitch in China is therefore based on a misidentification of *Aphis nerii*.

***Swirskiaphis bambuciepula* Zhang and Zhang should be in Rhopalosiphina**

*Swirskiaphis* was erected by Hille Ris Lambers (1966) for a species on Umbelliferae in western Asia related to *Aphis* but with long thick dorsal hairs. Zhang and Zhang (2000) described a second species in this genus, *Swirskiaphis bambuciepula* on *Phyllostachys* sp. (Gramineae) from Gansu Province, China. In this study, this species was embedded within Rhopalosiphina based on *COI* sequences. By checking the type of the species, and comparing with original description and figures of the type species, *Swirskiaphis polychaeta* Hille Ris Lambers (1966), some morphological features indicated the species should not be in genus *Swirskiaphis*. The dorsal setae on abdominal tergites are distinctly fewer, about half as long as in the type species; the processus terminalis is more than twice as long as the base of the last antennal segment; the marginal tubercles on abdominal tergite VII are placed above the level of spiracular pore; and the host plant is in a different family. Two very important diagnostic features, the position of the marginal tubercles on abdominal tergite VII and the length of the processus terminalis indicate that the species should be in Rhopalosiphina, not in Aphidina; and it seems likely that this species belongs in *Melanaphis*. Further studies with more specimens are needed in order to decide the generic placement of this species within Rhopalosiphina.

## Conclusion

Mean intraspecific sequence divergence in Aphidina was 0.52%, with a range of 0.00% to 2.95%, and the divergences of most species were less than 1%. The mean interspecific divergence with Aphidina was 6.80%, with a range of 0.68% to 11.40%, and most genera were in the range of 3.50% to 8.00%. A short *COI* sequence proved to be very useful for the identification of species within Aphidina. However, more specimens and DNA sequences are required to solve the remaining problems in classification and phylogeny.
